# A Rare Occurrence of Isolated Brain Metastases from Gastric Cancer

**DOI:** 10.1155/2019/8075421

**Published:** 2019-01-20

**Authors:** Manish M. Karamchandani, Tej Ganti, Sunny Jaiswal, Julian K. Wu, Muhammad Wasif Saif

**Affiliations:** ^1^Tufts University School of Medicine, Boston, MA 02111, USA; ^2^Department of Radiology, Tufts Medical Center, Boston, MA, USA; ^3^Department of Neurosurgery, Tufts Medical Center, Boston, MA, USA; ^4^GI Oncology Program, Hematology/Oncology, Tufts Medical Center, Boston, MA, USA

## Abstract

**Background:**

Gastric cancer is the fourth most common cancer worldwide and the second most common cause of cancer-related death. The majority of newly diagnosed gastric cancer cases present either as locally advanced tumor growth or with distant metastases.

**Case Report:**

Here, we describe a case of isolated brain metastases in a male patient with gastric cancer. Initially, our patient presented with dysphagia and was diagnosed with gastric cancer after a thorough evaluation. One year after chemotherapy and surgical resection of his gastric cancer, he presented with headaches, nausea, dizziness, and photophobia. Further evaluation of these symptoms led to the discovery of three metastatic brain lesions without evidence of extracranial metastases.

**Conclusions:**

Our review of the literature has found that such cases are rare. Additionally, our review of the literature demonstrates the poor outcomes associated with metastatic brain lesions from gastric cancer and highlights the importance of surgical resection in increasing overall survival time.

## 1. Introduction

Gastric cancer is the fourth most common type of cancer [1], with an incidence of nearly 1 million cases a year [2]. Nearly, 740,000 people worldwide die from gastric cancer each year, making it the second most common cause of cancer death [3]. In general, the incidence of gastric cancer is 2-3 times higher in men than in women [2]. The most common type of gastric cancer is adenocarcinoma, the etiology of which is multifactorial including diet, smoking, *H. pylori* infection, and medication/drugs such as NSAIDs. Surgery is the only curative option, with improved outcomes with neoadjuvant and adjuvant chemo/radiotherapy [1]. The majority of newly diagnosed gastric cancer cases involve locally advanced tumor growth or distant metastases [4]. Over 50% of radically resected gastric cancers recur either locally or with distant metastasis, or are diagnosed after the tumor has disseminated, leading to overall poor outcomes. The median survival is approximately 12 months, and the 5-year survival rate is less than 10% [1].

According to an analysis of a Swedish cancer registry, over 40% of patients presenting with gastric cancer have metastatic disease [5]. The most common sites of gastric cancer metastasis are the liver (48% of metastatic gastric cancer patients), peritoneum (32%), lung (15%), and bone (12%) [5]. Gastric cancer originating in the cardia of the stomach more frequently metastasizes to the lung, bone, and nervous system, while noncardia gastric cancer is more associated with peritoneum metastases [5].

In this case report, we describe a patient with gastric cancer who developed metastatic disease to the brain with multiple lesions without evidence of disease elsewhere. The incidence of such a case is <1% [6]. In the majority of gastric cancer cases with brain metastases, by the time brain metastases developed, there had been systemic spread to other organs. Approximately, half of patients with brain metastases will have multiple brain lesions [6].

## 2. Case Report

A 68-year-old Albanian man with history of chronic hepatitis B initially presented with progressive dysphagia to solids. Workup included a barium swallow showing a possible esophageal dysmotility problem and an abdominal ultrasound with no acute findings. The subsequent esophagogastroduodenoscopy demonstrated a nodule in the distal esophagus as well as a large, friable, and ulcerated mass along the gastric cardia extending into the gastroesophageal junction, both of which were biopsied. Pathology of the mass demonstrated adenocarcinoma, intestinal type, with neoplastic glands infiltrating the muscularis mucosae. Biopsy of the nodule showed high-grade dysplasia, and antral biopsies showed reactive mucosae. He was staged using positron emission tomography/computed tomography scans which confirmed the gastric mass and showed two fluorodeoxyglucose avid lymph nodes.

He underwent neoadjuvant chemotherapy with epirubicin, oxaliplatin, and capecitabine prior to total gastrectomy with esophagojejunal anastomosis. Following surgical resection, he was treated with paclitaxel, capecitabine, and pegfilgrastim kit.

**Figure 1:**
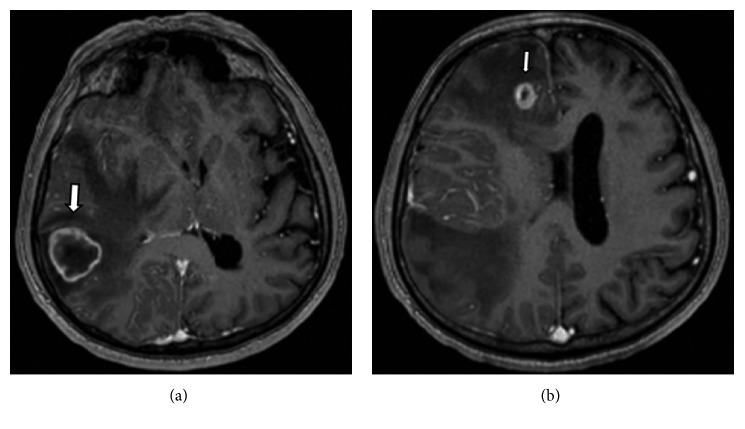
Contrast-enhanced T1-weighted images of the brain. (a) There is a ring enhancing approximately 3 cm mass (white thick arrow) in the right temporoparietal region with surrounding edema. This results in effacement of the right lateral ventricle and mild leftward midline shift. This was the dominate mass of the 3 lesions initially identified. (b) Similar smaller ring enhancing lesion (white thin arrow) in the right frontal lobe with surrounding edema.

One year later, he presented with a 3–6-week history of holocranial headaches and falls with dizziness, nausea, and photophobia. Magnetic resonance imaging of his head with and without contrast revealed three peripherally enhancing lesions with surrounding edema in his right cerebral hemisphere (Figures 1(a) and 1(b)). The largest lesion was in the right temporoparietal lobes, resulting in mass effect with midline shift. He underwent right temporoparietal craniotomy for resection of the largest tumor. The tumor stained positive for cytokeratin 7 (CK7) (cytoplasmic) and home box protein CDX-2 (nuclear) and was negative for keratin 20 (CK20), thyroid transcription factor 1 (TTF-1), human epidermal growth factor receptor 2 (HER-2) (+1/+3), and glial fibrillary acidic protein (GFAP), consistent with a gastrointestinal origin. He subsequently underwent gamma knife radiosurgery for the resection cavity and remaining two metastases, which were enlarged on MRI postcraniotomy. He was offered systemic chemotherapy including capecitabine with temazolamide but he declined and agreed to close monitoring. The patient remains to be stable both clinically and radiologically, with no new brain metastases and no evidence of metastases at any other site as of July 2018.

## 3. Discussion

Our patient presents a case of an elderly male with multiple brain metastases from gastric cancer without evidence of visceral metastases. There is a paucity of comparable information in the literature for several reasons: the relative rarity of gastric cancer in the U.S. when compared to more common gastrointestinal cancers such as colorectal cancer and the even smaller fraction of cases that present with brain metastases (in particular, parenchymal metastases as opposed to leptomeningeal carcinomas). Finally, this case is unusual as it is rare to find brain metastases from gastric cancer without any evidence of visceral metastases. From our review of the limited literature in Table 1, a majority of patients presenting with brain metastases from gastric cancer will have extracranial metastases (78.2%). Other demographic information from our review of the literature as shown in Table 1 demonstrates that brain metastases from gastrointestinal cancers are most common in East Asian males (75.5%), consistent with the demographic profile of gastric cancer, whereas our patient is an Albanian male. Approximately, half of patients with brain metastases will have more than one brain lesion (50.4%).

Due to the limited number of individually reported cases that underwent surgical resection, evaluating the effectiveness of surgical resection is best achieved through a review of multipatient analyses, as presented in Table 1. By weighting each study for the number of included patients, the average overall survival for gastric cancer patients with brain metastases was 5.6 months. However, for patients that underwent surgical resection, the overall survival was over twice as long (11.4 months). Our patient had a combination of chemotherapy and radiation therapy with surgical resection. The patient is still alive as of July 27, 2018, with excellent performance status and no new brain metastases on the most recent MRI of the head, 10 months after the initial diagnosis of brain metastases.

The improved overall survival with surgical resection could reflect a better initial prognosis compared to patients who received chemotherapy, radiotherapy, or only palliative care. However, closer examination of the data in Table 1 suggests otherwise. For example, the study by Jun et al. is notable for having a relatively low percentage of patients with extracranial metastases (44%) and multiple brain metastases (11%), yet the percent of patients who underwent resection was 22%, close to the average (20.9%) across the various analyses. Furthermore, the study by York et al. had above average numbers of patients with extracranial metastases (88%) and multiple brain metastases (55%), yet the overall survival was still markedly improved with resection (12.5 months compared to 2.4 without resection).

A review of the case reports described in Table 2 reveals a wide variety of presentations and treatment options, including not only surgical resection but also chemotherapy and various forms of radiotherapy. Furthermore, although the individual cases demonstrated a similar male prevalence to the clinical analyses in the literature, they had on average fewer extracranial metastases (50% vs 78.2%) and fewer cases with multiple brain metastases (29% vs 50.4%). This could account for the higher average survival time from diagnosis (16.6 months average in the individual cases compared to 5.6 months in the clinical analyses), also seen in the patient who underwent resection (16.4 months vs 11.6 months). The study of 9 patients by Jun et al. also featured a comparable survival time of 16 months in a patient population with lower percentages of extracranial metastases and multiple brain metastases. In addition, the similar survival times with and without resection (16.6 months vs 16.4 months) in the individual cases could reflect a decreased effectiveness of surgical resection in patients with more limited symptomology, especially since the individual cases reveal a higher percentage of patients who underwent resection (50% vs 20.9% in the clinical analyses). However, there are only a few individual resection cases with considerable variability in results (survival times ranging from 2 months to 5 years), indicating that more data are required before drawing additional conclusions.

Overall, when patients are symptomatic from their metastases, surgical resection should be considered. Surgical resection appears to increase patient survival, based on the published case series. However, as these are small retrospective studies, confounding variables such as patient selection and treatment bias can influence results. Additional research is required to eliminate confounding variables such as alternative treatments while considering secondary patient outcomes such as quality of life.

## Figures and Tables

**Table 1:** Clinical analyses of survival and characteristics in gastric cancer patients diagnosed with brain metastases.

*n*	Median age (years)	% male	% ECM	% BM > 1	% resection	Median OS (months)	Median OS resection (months)	Source
24	53	75	88	55	21	2.4	12.5	York et al. [7]
11	44	54	n/a	n/a	0	1.8	n/a	Kim [8]
11	55	82	20	55	27	2.7	6	Kasakura et al. [9]
11	61	64	82	45	18	27.7	45.5	Han et al. [10]
56	56	79	91	55	n/a	5.7	n/a	Park et al. [11]
9	74	77	44	11	22	16	n/a	Jun et al. [12]
4	76	100	n/a	n/a	50	2	n/a	Tamura et al. [13]
67	59	77	n/a	n/a	18	3.5	6.2	Lee et al. [14]
16	68.5	69	75	50	37.5	2.8	12.3	Kraszkiewicz and Wydmanski [15]
	**58.3**	**75.5**	**78.2**	**50.4**	**20.9**	**5.6**	**11.4**	*Weighted average*

*n* = number of patients enrolled in the study; male = percentage of male patients of the patient cohort; ECM = percentage of patients diagnosed with extracranial metastases; BM > 1 = percentage of patients diagnosed with more than one brain metastases; resection = percentage of patients that received surgical resection of brain metastases; OS = median survival of all patient diagnosed with brain metastases within the respective study; OS resection = median survival of the patients who received surgical resection of brain metastases; n/a = data not available. All sources are parenchymal brain metastasis cases, not leptomeningeal carcinomas.

**Table 2:** Individual case reports of survival and characteristics in gastric cancer.

Age (years)	% male	% ECM	% BM > 1	% resection	OS (months)	OS resection (months)	Source
61	100	0	0	0	10	n/a	Yang et al. [16]
78	0	100	0	0	5	n/a	Sakurai et al. [17]
74	0	0	0	100	2	2	Philip et al. [18]
68	100	100	0	100	60	60	Nakazawa et al. [19]
49	100	100	100	0	24	n/a	Kojima et al. [20]
57	100	0	0	100	10	10	Joo et al. [21]
76	100	100	100	100	9	n/a	Kitayama et al. [22]
47	100	100	0	0	4	n/a	Nomura et al. [23]
51	100	0	0	0	12	n/a	Hizawa et al. [24]
51	100	0	100	100	18	18	Murawa et al. [25]
47	100	100	0	100	6	6	Perri et al. [26]
53	0	100	100	0	0.2	n/a	Sakaki et al. [27]
64	100	0	0	100	2.5	2.5	Nakabayashi et al. [28]
73	100	0	0	0	70	n/a	Peng et al. [29]
**60.6**	**79**	**50**	**29**	**50**	**16.6**	**16.4**	*Average*

Age = age in years at time of brain metastasis diagnosis; male = percentage of male patients of the patient cohort; ECM = percentage of patients diagnosed with extracranial metastases; BM > 1 = percentage of patients diagnosed with more than one brain metastases; resection = percentage of patients that received surgical resection of brain metastases; OS = median survival of all patient diagnosed with brain metastases within the respective study; OS resection = median survival of the patients who received surgical resection of brain metastases; n/a = data not available. All sources are parenchymal brain metastasis cases, not leptomeningeal carcinomas.
